# Vaginal Adenocarcinoma: A Review of a Rare Gynecologic Cancer

**DOI:** 10.3390/cancers17132130

**Published:** 2025-06-25

**Authors:** Mun-Kun Hong, Dah-Ching Ding

**Affiliations:** 1 Department of Obstetrics and Gynecology, Hualien Tzu Chi Hospital, Buddhist Tzu Chi Medical Foundation, Tzu Chi University, Hualien 970, Taiwan; jeff06038@gmail.com; 2 Institute of Medical Sciences, Tzu Chi University, Hualien 970, Taiwan

**Keywords:** vagina, cancer, adenocarcinoma, rare, treatment, immunotherapy

## Abstract

Background/Objectives: Vaginal adenocarcinoma is a rare malignancy, accounting for less than 10% of all primary vaginal cancers. It predominantly affects older women but can also occur in younger populations, particularly in association with in utero diethylstilbestrol (DES) exposure. Given its rarity, evidence regarding the optimal management of vaginal adenocarcinoma remains limited. This review aimed to summarize the current understanding of vaginal adenocarcinoma, covering the epidemiology, etiology, diagnostic approaches, treatment modalities, prognosis, and areas requiring further investigation thereof. Methods: We conducted a search for the term “vaginal adenocarcinoma” in the PubMed, Scopus, and Web of Science databases from January 2016 to 28 April 2025. Results: Overall, 83 articles were included in the final review. Among them, 21 cases of vaginal adenocarcinoma were reported. Vaginal adenocarcinoma demonstrates a bimodal age distribution, with clear cell histology commonly linked to DES exposure and endometrioid or mucinous types seen in older patients. Risk factors include DES exposure, chronic inflammation, and human papillomavirus (HPV) infection. The diagnosis relies on a pelvic examination, imaging, and biopsy. Treatment typically involves surgery, radiotherapy, or a combination thereof, tailored to the stage and location, with chemotherapy reserved for advanced cases. The prognosis depends on the histologic subtype, tumor size, stage, and treatment response, with early-stage disease generally associated with better outcomes. Conclusions: Improved awareness of risk factors and early diagnostic strategies is critical to optimize patient outcomes. Research is needed to refine treatment protocols, explore targeted therapies and immunotherapy, and investigate the molecular underpinnings of vaginal adenocarcinoma, particularly non-DES-associated types.

## Simple Summary

Vaginal cancer is rare and represents less than 10% of primary vaginal cancers, with cases linked to in utero diethylstilbestrol (DES) exposure or occurring sporadically in older women. This review, based on the PRISMA guidelines, analyzed 21 case reports from 83 eligible studies published between 2016 and 2025. Vaginal cancer shows a bimodal age distribution, with clear cell types common in DES-exposed patients and endometrioid or mucinous types in older adults. Risk factors include DES exposure, chronic inflammation, and human papillomavirus (HPV) infection. Vaginal cancer is diagnosed using a pelvic exam, imaging, and biopsy, while treatment is stage-dependent, often combining surgery and radiotherapy. The prognosis varies by the histologic type, size, and stage, with early-stage disease faring better. Greater awareness, early detection, and further research into tailored therapies and molecular mechanisms are essential to improve patient care.

## 1. Introduction

Vaginal cancer is a rare gynecological malignancy, accounting for approximately 3% of all gynecologic cancers [1,2]; the primary site of growth thereof is in the vagina, excluding secondary growths from genital or extra-genital sites [3]. Squamous cell carcinoma is the most common histological type, followed by adenocarcinoma [1,2]. Additionally, the Federation Internationale de Gynecologie et d’Obstetrique (FIGO) staging system for vaginal cancer follows similar rules to cervical cancer and is clinically staged [1].

Mesonephric adenocarcinoma of the vagina, arising from mesonephric remnants, is particularly uncommon [4]. Historically, vaginal clear cell carcinoma was associated with prenatal diethylstilbestrol (DES) exposure; however, cases without such exposure have been reported [5]. Risk factors for vaginal cancer include HPV infection, particularly type 16, as well as sexual behavior, chronic vaginitis, and vaginal chemical exposure [6]. The diagnosis of vaginal cancer can be challenging, requiring clinical, radiological, and immunohistochemical correlations to differentiate primary from metastatic tumors [7]. In utero DES exposure is also associated with some cases, particularly in younger patients [8].

Treatment approaches for vaginal cancer vary and include surgery, radiotherapy, and chemotherapy, with surgical excision showing promising outcomes [7,8]. Treatment primarily involves radiation therapy, with surgery considered on an individual basis [2]. Recent studies have also explored the benefits of chemoradiation for advanced-stage disease [3]. The prognosis of vaginal cancer depends on factors such as age, the tumor stage, and metastasis status [7]. Five-year survival rates for patients with early-stage (I or II) vaginal cancer can reach 80–90% with treatment, while the survival rates for patients with advanced disease remain at approximately 30% [2]. This study aimed to review the clinicopathologic features, immunohistochemical profiles, and differential diagnostic considerations of primary vaginal adenocarcinomas, with an emphasis on distinguishing them from metastatic tumors and other gynecologic malignancies through molecular and immunohistochemical markers.

## 2. Materials and Methods

### Search Strategy

A systematic search was conducted of the PubMed, Scopus, and Web of Science databases using the keyword “vaginal adenocarcinoma”. The search covered studies from the inception of each database or from January 2016 to 7 April 2025. Synonyms and related terms for the keywords were also included. Additionally, the reference lists of relevant reviews and selected studies were examined. Table 1 outlines the detailed search strategy employed for each database.

## 3. Results

### 3.1. Screening Results

**Figure 1:**
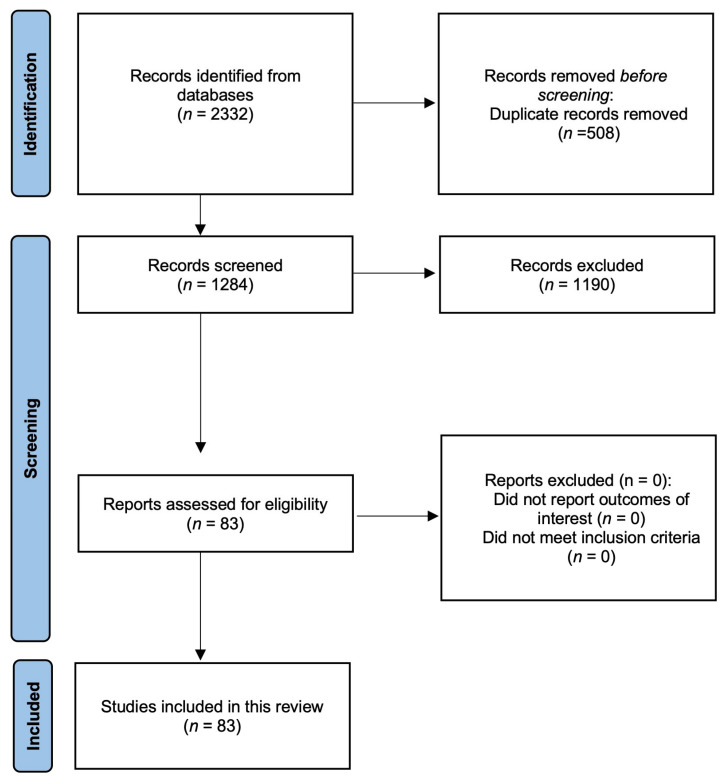
Algorithm for the selection of articles in the literature review.

Initially, 2332 articles were extracted from the databases, of which 508 were removed because of duplication. The remaining 1284 articles were reviewed based on their titles and abstracts, and 1190 were removed because of irrelevance. Subsequently, 83 articles were reviewed based on our exclusion criteria, and zero were excluded. Finally, 83 articles met the inclusion criteria and were included in the review (Figure 1).

### 3.2. Literature Review

Initially, 83 articles met the general inclusion criteria for full-text review. After applying detailed case-specific criteria, 21 articles describing 21 individual cases of vaginal adenocarcinoma were included in the final synthesis.

A comprehensive summary, including age, clinical presentation, diagnosis, management, and outcomes, is presented in Table 2.

A review of 21 reported cases of primary vaginal adenocarcinoma and related rare vaginal malignancies highlights the diversity in patient demographics, clinical presentations, histologic subtypes, and treatment approaches. Patient ages ranged widely, with cases involving both young women and the elderly. Common presenting symptoms included vaginal bleeding, palpable masses, pelvic pain, and recurrent discharge. Histologic diagnoses encompassed clear cell carcinoma, mesonephric and endometrioid adenocarcinomas, intestinal-type adenocarcinoma, adenoid cystic carcinoma, HPV-associated enteric-type adenocarcinoma, and rare entities such as small-cell neuroendocrine carcinoma and cystadenocarcinoma of the rectovaginal septum. Management strategies varied depending on the disease stage and histology, including surgery, radiotherapy, chemotherapy, immunotherapy (notably pembrolizumab), and in some cases, advanced techniques like proton beam therapy or fluorescence-guided surgery. While several patients achieved complete responses and long-term survival, others had poor prognoses, especially those with advanced stages or aggressive subtypes. These findings underscore the need for individualized, multidisciplinary treatment and continued documentation of rare presentations to guide optimal management.

## 4. Discussion

### 4.1. Epidemiology

#### 4.1.1. Global and Regional Incidence and Prevalence

Primary vaginal carcinoma is uncommon, accounting for only 1–2% of all gynecological malignancies, and mesonephric adenocarcinoma represents less than 0.1% of vaginal cancers [28].

#### 4.1.2. Demographics (Age Groups, Racial/Ethnic Differences)

Vaginal adenocarcinoma is most commonly diagnosed in women aged 30 or younger, in contrast to squamous cell carcinoma, which typically affects women aged 60 and older [29]. Clear cell adenocarcinoma—a distinct subtype—peaks in incidence between ages 17 and 21 and is frequently associated with in utero exposure to diethylstilbestrol (DES) [8]. Although the median age at diagnosis for all vaginal cancers is approximately 67–68 years, adenocarcinoma is notably more prevalent among younger women, particularly those under 30 years. Vaginal cancer, including adenocarcinoma, has the highest incidence among non-Hispanic Black women compared to other racial and ethnic groups, although the overall incidence remains low across all groups. The prevalence per 100,000 people for all vaginal cancers is: non-Hispanic Black (0.87), non-Hispanic White (0.64), Hispanic (0.64), non-Hispanic Asian/Pacific Islander (0.37), and non-Hispanic American Indian/Alaska Native (0.00). Additionally, Black, Asian/Pacific Islander, and Hispanic women are more likely to be diagnosed at a later stage and experience lower 5-year survival rates than white and non-Hispanic women [30].

#### 4.1.3. Risk Factors and Associated Conditions

DES Exposure: In utero exposure to DES, a synthetic estrogen prescribed to pregnant women from the 1940s to early 1970s to prevent miscarriage, is the most specific and significant risk factor for vaginal adenocarcinoma, particularly the clear cell subtype. The risk is highest when exposure occurs during the first 16 weeks of gestation [31].

Age: While most vaginal cancers develop in older women, clear cell adenocarcinoma is more commonly seen in younger women, especially those with a history of prenatal DES exposure [32].

Human Papillomavirus (HPV) Infection: Persistent infection with high-risk HPV types is a well-established risk factor for vaginal cancer, particularly squamous cell carcinoma. Although less strongly associated, HPV may also contribute to some cases of adenocarcinoma [33].

Immunosuppression: Conditions that impair immune function, such as HIV infection, increase the risk of vaginal cancer, including adenocarcinoma, by reducing the body’s ability to clear oncogenic viruses like HPV [33].

Smoking: Tobacco use more than doubles the risk of vaginal cancer, including adenocarcinoma, particularly in women with HPV infection [33].

### 4.2. Histological Subtypes and Etiology

#### 4.2.1. Clear Cell Adenocarcinoma (Most Common Type)

The most common variant of vaginal adenocarcinoma is clear cell adenocarcinoma, characterized by cells with clear cytoplasm and hobnail morphology, often arranged in tubulocystic, papillary, or solid patterns. Clear cell carcinoma is strongly associated with prenatal DES exposure, though sporadic cases unrelated to DES occur, which can arise spontaneously or in women with in utero exposure to DES [34,35,36,37]. Primary non-DES-related vaginal adenocarcinoma is rare and predominantly affects postmenopausal women. DES was widely prescribed in the late 1940s and early 1950s to support high-risk pregnancies, particularly in women with a history of miscarriage, diabetes, or multiple gestations [38,39,40]. Approximately 5% of all pregnant women in the United States during that period were treated with DES. Herbst et al. described seven young women (aged 15–22 years) treated at Vincent Memorial Hospital (Boston, MA, USA) between 1966 and 1969 who developed clear cell or endometrioid-type adenocarcinomas associated with in utero DES exposure [41].

#### 4.2.2. Mesonephric Adenocarcinoma

Mesonephric adenocarcinoma originates from Wolffian duct remnants, typically located in the lateral vaginal wall, and exhibits a variety of architectural patterns, including compact tubules, solid sheets, endometrioid-like glands, and cribriform structures [12]. The presence of hyaline eosinophilic intraluminal secretions is considered pathognomonic. Mesonephric adenocarcinoma characteristically expresses mesonephric markers such as GATA3, TTF1, and PAX2, while lacking hormone receptor expression. Studies have identified KRAS mutations—most commonly p.G12D—in approximately 60% of cases, along with rare TP53 mutations associated with aberrant p53 expression [4]. In contrast to clear cell adenocarcinoma, mesonephric adenocarcinoma is not associated with DES exposure or human papillomavirus infection.

#### 4.2.3. Endometrioid Adenocarcinoma

A rare subtype, accounting for less than 2% of vaginal adenocarcinomas, endometrioid adenocarcinoma resembles uterine endometrioid carcinoma, featuring a glandular architecture and often squamous metaplasia [13]. This tumor is HPV-independent and may originate from endometriosis or vaginal adenosis [14].

#### 4.2.4. Serous Carcinoma

Serous carcinoma is characterized by papillary structures and high-grade nuclei; this subtype is typically metastatic, most often arising from fallopian tube or ovarian primaries. Primary vaginal serous carcinoma is exceedingly rare [42].

#### 4.2.5. HPV Involvement

While high-risk HPV types 16 and 18 are well-recognized drivers of vaginal squamous cell carcinoma, their involvement in vaginal adenocarcinoma is far less common and remains poorly characterized [43]. A handful of case reports and small series have, however, documented HPV-associated vaginal adenocarcinomas. These tumors frequently exhibit strong p16 overexpression and, in some instances, harbor detectable high-risk HPV DNA [44].

### 4.3. Clinical Presentation

#### 4.3.1. Symptoms

The clinical presentation of vaginal adenocarcinoma closely resembles that of other histologic types of vaginal cancer, with symptoms typically arising from local tumor growth and invasion (Table 3). Key presenting features include the following:

Abnormal vaginal bleeding—the most common symptom, occurring in 50–75% of cases, often presenting as postmenopausal bleeding, postcoital bleeding, or intermenstrual spotting [10,45].

Vaginal discharge—patients may experience a persistent or malodorous discharge, potentially indicating tumor necrosis or secondary infection [10,46].

Palpable mass or lump—a nodule or area of thickening may be palpable, particularly in the upper third of the vaginal wall [17].

Pain or discomfort—pelvic pain, dyspareunia (pain during intercourse), or rectal pain may develop, especially as the disease advances [24,46].

Other symptoms—less commonly, patients may report pruritus (itching), burning sensations, urinary symptoms (such as dysuria, hematuria, or increased frequency), constipation, or hematuria if adjacent organs are involved [26].

#### 4.3.2. Physical Examination Findings

Vaginal adenocarcinomas may present as either exophytic (fungating) or ulcerative lesions [34]. Clear cell adenocarcinoma is often associated with vaginal wall thickening and rigidity due to submucosal infiltration [26]. While more than half of primary vaginal cancers are located on the posterior wall of the upper third of the vagina, adenocarcinomas can arise at any site within the vaginal canal [47].

#### 4.3.3. Patterns of Spread (Local Invasion and Lymphatic Spread)

The local invasion and lymphatic spread patterns encompass the following aspects:

Direct extension—Vaginal tumors can invade adjacent pelvic soft tissues, including the paravaginal tissue, parametria, urethra, bladder, and rectum. Most tumors originate in the upper third of the vagina, particularly along the posterior wall.

Lymphatic spread—Lymphatic drainage of the vagina is complex. The upper vagina drains primarily to the pelvic lymph nodes, namely, the obturator, internal iliac (hypogastric), and external iliac nodes. Involvement of the para-aortic nodes is rare. The lower vagina drains to the inguinal and femoral (groin) nodes. Tumors located in the midvagina may spread to both pelvic and groin nodes [48].

Hematogenous spread—Distant metastases via hematogenous spread to the lungs, liver, and bone typically occur in advanced stages.

### 4.4. Diagnostic Evaluation

Diagnosis typically involves imaging and biopsy, while treatment options include surgery, chemotherapy, and radiotherapy [12]. Molecular characterization has revealed KRAS mutations in MA, with rare cases harboring TP53 mutations [4].

#### 4.4.1. Pelvic Examination and Colposcopy

During a pelvic examination, vaginal adenocarcinoma may manifest as a visible lesion, frequently located on the posterior vaginal wall. Examiners should be vigilant for signs such as an abnormal discharge, tissue irregularities, or palpable masses within the vaginal canal. Notably, lesions on the posterior wall can sometimes be obscured by a speculum, underscoring the importance of a thorough inspection. In cases of intestinal-type adenocarcinoma, which predominantly affect the lower third of the vagina, careful examination of this area is crucial, as small lesions may be missed during initial inspections due to their location [49].

Colposcopy is a valuable diagnostic tool utilized to evaluate vaginal adenocarcinoma, particularly the intestinal-type, which frequently involves the posterior wall and lower third of the vagina [49]. By providing a magnified view of the vaginal epithelium, colposcopy enables clinicians to detect subtle mucosal abnormalities that may be overlooked during routine pelvic examinations. This enhanced visualization facilitates targeted biopsies of suspicious areas, aiding in an accurate diagnosis and informing appropriate treatment strategies.

An examination under anesthesia may aid in confirming the diagnosis, obtaining sufficient tissue for histologic evaluation and comprehensive molecular profiling (e.g., PD-L1), and assessing the extent of disease [50]. Physicians should consider performing cystoscopy and proctoscopy concurrently to rule out bladder or rectal invasion [51]. Additionally, an evaluation of the cervix and vulva should be performed to exclude other gynecologic primary sites.

#### 4.4.2. Imaging Studies

Magnetic resonance imaging (MRI) is the preferred modality for local staging of gynecologic cancers due to its superior soft-tissue contrast, enabling precise assessments of the tumor size, depth of invasion, and involvement of adjacent structures [52]. The FIGO 2018 guidelines recommend incorporating imaging modalities such as MRI or positron emission tomography (PET)/computed tomography (CT) alongside pelvic examinations for comprehensive staging of gynecologic malignancies [52]. Integrated PET/MRI combines metabolic information from PET with the anatomical detail of MRI, offering enhanced diagnostic performance. In cervical cancer staging, PET/MRI has demonstrated higher accuracy compared to CT and MRI alone, particularly in detecting lymph node metastases and assessing the local tumor extent. While PET/MRI show promise in integrating metabolic and anatomical data for gynecologic cancer staging, further research is ongoing to establish their definitive roles in clinical practice [53].

#### 4.4.3. Immunohistochemical Profiles and Differential Diagnosis

Immunohistochemistry (IHC) is pivotal in distinguishing primary vaginal adenocarcinomas from metastatic lesions and in subtyping primary tumors (Table 4). Below, we detail the clinical significance of positive and negative markers, including approximate expression changes observed in specific adenocarcinomas.

##### Intestinal-Type Vaginal Adenocarcinoma

Positive markers include the following:

- SATB2—highly sensitive for colorectal origin (>90% expression), and its expression increases 2- to 5-fold in intestinal-type tumors compared to Müllerian or cervical adenocarcinomas;
- CDX2—nuclear staining is strong (>80% of cases), and its expression levels are 3- to 10-fold higher than in non-intestinal adenocarcinomas;
- CK20—typically positive (70–90% of cases), and its expression is markedly elevated (5- to 20-fold) compared to gynecologic tumors;
- CEA—cytoplasmic positivity in >70% of cases, and its levels may rise 2- to 4-fold in metastatic intestinal-type tumors [54].

Negative markers (clinically significant reductions) include the following:

- CK7—absent or faint (<5% of cases), and its expression is reduced by >90% compared to Müllerian tumors;
- PAX8—negative (≤1% of cases), and near-undetectable levels help exclude a Müllerian origin;
- p16—patchy or negative (HPV-independent), and its expression is 50–80% lower than in cervical adenocarcinomas;
- GATA3—negative and absent in intestinal-type tumors but strongly expressed in urothelial/breast cancers [55].

Key Roles: SATB2/CDX2 confirm intestinal differentiation, while CK7/PAX8 exclusion is critical to rule out gynecologic primary tumors.

##### Endometrial and Ovarian Adenocarcinomas

Positive markers include the following:

- PAX8—strong nuclear positivity (>95% of cases), and its expression is 5- to 50-fold higher than in colorectal tumors;
- CK7—diffuse cytoplasmic staining (>90%) that is 10- to 30-fold higher than CK20 in these tumors;
- ER/PR—hormone receptors are 2- to 10-fold more abundant in endometrioid subtypes vs. serous/cervical tumors [56].

Negative markers include the following:

- SATB2—negative (≤5% of cases), with a >95% reduction compared to colorectal tumors;
- CK20—rarely expressed (<10%), and the levels are >80% lower than in gastrointestinal adenocarcinomas [56].

Note: PAX8’s high sensitivity for a Müllerian origin is tempered by its rare expression in endocervical tumors (typically 50% lower intensity).

##### Cervical Adenocarcinomas

Positive markers include the following:

- p16—diffuse strong positivity (HPV-related), and its expression increases 10- to 100-fold in high-risk HPV-associated tumors;
- CEA—focal to diffuse (60–80% of cases) and its levels may rise 2- to 5-fold in endocervical primaries.

Negative markers include the following:

- ER/PR—Negative or weak (<10% of cases), with a >90% reduction compared to endometrial tumors;
- Vimentin—typically absent and >70% lower than in endometrial carcinomas [7].

Key Role: p16’s dramatic overexpression distinguishes HPV-driven cervical tumors from endometrial mimics.

##### Serous Papillary Adenocarcinomas

Positive markers include the following:

- PAX8/WT1—strong nuclear staining (>90%), and WT1 expression is 5- to 20-fold higher than in non-serous tumors;
- p53—aberrant (overexpressed/null) in >80% of cases, and mutant p53 levels may be 10- to 50-fold higher than wild-type levels;
- CA125—elevated in 70–90% of cases, with serum levels often 100-fold above normal in advanced disease [54].

Negative markers include the following:

- SATB2/CK20—negative; its expression is >95% lower than in gastrointestinal tumors [57].

Key Roles: WT1/p53 co-expression confirms serous differentiation, while SATB2 exclusion is critical for ruling out metastatic CRC.

#### 4.4.4. Staging (FIGO Classification for Vaginal Cancer)

The 2009 FIGO staging for primary vaginal cancer is described and compared with other systems in Table 5 [46].

### 4.5. Management Strategies

#### 4.5.1. Surgery

Surgery is a primary treatment for vaginal adenocarcinoma, particularly early-stage disease. The choice of surgical approach depends on factors such as the tumor stage, size, location within the vagina, and lymph node involvement [58]. According to FIGO guidelines, for stage I adenocarcinoma (tumors < 2 cm), especially those located in the upper vagina, the standard surgical treatment includes a radical hysterectomy, partial or total vaginectomy, and pelvic lymph node dissection [46,59,60].

The lymph node assessment is guided by the tumor location [58] as follows: tumors in the upper two-thirds of the vagina typically drain to pelvic lymph nodes, necessitating pelvic lymphadenectomy; tumors in the lower one-third of the vagina often involve inguinal lymph nodes, requiring inguinal lymphadenectomy; for microscopic lesions at the apex of the vagina, an upper vaginectomy with or without hysterectomy may be appropriate, for which the margins are likely to be negative. Every effort should be made to obtain negative margins.

#### 4.5.2. Radiation Therapy

According to FIGO guidelines, radiotherapy using external beam radiation therapy (EBRT), brachytherapy, or a combination of both is the standard treatment for patients with stage II–IVA vaginal cancer, particularly locally advanced cases [46,61,62,63]. The primary advantage of radiation therapy is the preservation of organ function. A systematic review by Guerri et al. identified several factors associated with improved outcomes for patients receiving radiotherapy, including early-stage disease, a tumor size <4 cm, prior hysterectomy, higher pre-treatment hemoglobin levels, and a younger age [63]. Two additional retrospective studies also reported excellent outcomes with definitive radiotherapy, whether administered as EBRT alone or in combination with brachytherapy, highlighting the importance of tailoring radiotherapy to individual patient characteristics [64,65]. With recent advancements in radiation technology, image-guided radiotherapy is increasingly employed in the treatment of vaginal cancer, offering enhanced precision, reduced exposure to surrounding healthy tissue, and lower rates of toxicity [66].

For very early-stage vaginal cancers (<5 mm in depth) that do not require EBRT, intracavitary brachytherapy alone may be an appropriate treatment option [67]. Data from low dose-rate brachytherapy suggest improved outcomes with doses of approximately 60–70 Gy EQD2 administered to the vaginal surface. High dose-rate (HDR) data are more variable, with total doses typically ranging from 50 to 60 Gy EQD2. The dose selection should be individualized based on tumor characteristics and patient factors [67]. Common HDR regimens include 5 Gy × 8 fractions or 8 Gy × 5 fractions, delivered to the vaginal surface twice weekly [67].

#### 4.5.3. Chemoradiotherapy

Chemotherapy is rarely used as a standalone treatment for vaginal cancer; instead, it is combined with other modalities [46]. Combined approaches and clinical decision-making on concurrent chemoradiotherapy (CCRT) enhance the treatment outcomes of patients with stage II–IV vaginal cancer. A large retrospective cohort study involving 8222 patients demonstrated that chemoradiation was associated with a significant improvement in median overall survival compared to radiation therapy alone [68]. Similarly, a single-institution study of 71 patients identified concurrent chemotherapy as a significant predictor of improved disease-free survival [69]. Treatment decisions for vaginal cancer remain poorly defined, particularly for patients with an intact uterus, and even more so for those who have undergone hysterectomy. In our review, treatment approaches were heterogeneous, lacking a standardized strategy. However, insights can be drawn from a recent case we encountered. A patient with stage I endometrioid vaginal cancer located in the vaginal cuff underwent complex surgical resection, which was associated with significant blood loss and surgical difficulty. Even in the hands of an experienced gynecologic oncologist, surgical management of post-hysterectomy patients proved to be highly challenging and carried a substantial risk of complications.

The standard CCRT regimen typically involves the use of cisplatin (75 mg/m^2^ on day 1) combined with 5-fluorouracil (5-FU) (1000 mg/m^2^/day, days 2–5). A study of 14 patients (71% stage II/III) treated with 5-FU-based CCRT showed promising local control and remained a significant predictor of disease-free survival (DFS) (hazard ratio 0.31, 95% confidence interval [CI], 0.10–0.97; *p* = 0.04) [69]. In a patient with advanced vaginal cancer (stage IVB), complete remission was achieved following CCRT with 5-FU and cisplatin, with no evidence of recurrence at 40-month follow-up and a remission duration of 20 months [70]. For patients who are unable to tolerate cisplatin due to factors such as renal dysfunction or other comorbidities, carboplatin serves as an alternative.

#### 4.5.4. Emerging Targeted Therapies or Immunotherapy

When recurrence occurs, patients whose tumors express PD-L1 are recommended to use checkpoint inhibitors and/or monoclonal antibodies [71].

### 4.6. Prognosis

The prognosis of vaginal adenocarcinoma is influenced by several factors, with the stage at diagnosis being the most critical determinant. Recent data indicate a 5-year relative survival rate of approximately 60% for adenocarcinomas, though this varies widely depending on individual circumstances [72].

#### 4.6.1. Key Prognostic Factors

For vaginal cancers in general, an HPV-positive status (and p16 positivity) is associated with a better prognosis compared to HPV-negative tumors [73]. HPV-associated tumors may respond better to immune checkpoint inhibitor therapies, reflecting a more immunogenic tumor microenvironment [74].

#### 4.6.2. Stage at Diagnosis

Early-stage (I/II) vaginal adenocarcinomas are associated with more favorable outcomes, largely due to the effectiveness of localized treatment [75]. In contrast, advanced stages (III/IV) have markedly lower survival rates. For example, stage IV vaginal cancers, across all histologic types, have 5-year survival rates ranging from 18% to 36% [72].

#### 4.6.3. Histologic Comparison

In subgroups such as clear cell adenocarcinoma linked to prenatal DES exposure, adenocarcinomas may demonstrate a slightly better prognosis than squamous cell carcinoma. However, other studies suggest that overall, adenocarcinomas may have poorer outcomes [75], reflecting differences in tumor biology and responsiveness to treatment.

#### 4.6.4. Impact of Treatment

Surgical intervention is associated with improved survival, with a 5-year survival rate of 72.1% reported among patients undergoing surgery for vaginal cancer of any type [72]. Radiation therapy also significantly improves outcomes compared to no treatment [72].

#### 4.6.5. Additional Influencing Factors

A tumor size greater than 4 cm is linked to a notable decrease in survival [75]. An age 80 years or older is also associated with worse outcomes. While not directly applicable to adenocarcinoma, melanoma histology serves as an example of how the tumor subtype can profoundly impact the prognosis [72].

#### 4.6.6. Recurrence Patterns and Follow-Up Strategies

##### Recurrence Patterns

- The recurrence pattern of vaginal adenocarcinoma is highly variable, with no consistent trend. In a study of 320 patients treated with radical vaginal trachelectomy (RVT), 10 (3.1%) experienced recurrence at a mean of 26.1 months post-treatment, despite the absence of identifiable high-risk factors. Recurrence may be local (vaginal vault or cervix), regional (pelvic lymph nodes or adjacent organs), or distant (lungs, liver, or bones) [76].
- Clear cell adenocarcinoma has an overall recurrence rate of ~21%, with a predilection for the lungs, supraclavicular lymph nodes, and pelvis [75].
- Distant metastases in adenocarcinomas frequently involve the lungs, liver, adrenal glands, and bones, with higher rates of peritoneal carcinomatosis compared to squamous cell carcinomas [77].

##### Survival Rates and Kaplan–Meier Data

While our study did not generate original Kaplan–Meier curves due to cohort heterogeneity, the aggregated literature suggests the following:

- The 5-year overall survival (OS) for early-stage (I–II) vaginal adenocarcinoma ranges from 65–80%, declining to <30% for advanced stages (III–IV) [72];
- Disease-free survival (DFS) at 3 years is ~60–70% for localized disease but drops to <20% with distant metastasis [78,79];
- For the clear cell subtype, a Registry for Research on Hormonal Transplacental Carcinogenesis (*n* = 695) reported a 5-year OS of 86.1% for patients with prenatal DES exposure and 81.2% for patients without DES exposure [80].

##### Imaging and Prognosis

MRI and PET/CT are critical for detecting recurrence, as early intervention (e.g., chemoradiation) improves median survival from 6–12 months (untreated metastasis) to 18–24 months with multimodal therapy [77].

##### Follow-Up Strategies

- Given the unpredictable recurrence and poor prognosis of metastatic disease, close surveillance is essential.
- The frequency of clinical exams and imaging (e.g., MRI) is every 3 months for the first 2 years, and then biannually up to 5 years [76].
- For high-risk cases, consider tumor markers (e.g., CA125 for serous subtypes) and PET/CT for suspected relapse [77].

### 4.7. Special Considerations

#### 4.7.1. Young Patients with DES-Related Clear Cell Carcinoma

Exposure to DES is strongly linked to the development of clear cell carcinoma of the vagina, especially in young women during their second decade of life [81]. The drug was banned, but DES-exposed daughters were reported to have an increased risk of clear cell adenocarcinoma persisting into older ages and are at increased risk for melanoma at young ages, but not other cancers [82].

#### 4.7.2. Fertility Preservation

In patients who wish to remain fertile, ovarian preservation or transposition should be considered when feasible [83]. In select cases, fertility-sparing procedures, such as radical abdominal trachelectomy combined with upper vaginectomy, have been successfully performed in young women with early-stage disease [84].

#### 4.7.3. Psychosocial Impact and Quality of Life

Vaginal cancer can have a profound impact on patients’ psychosocial well-being and overall quality of life (QoL), affecting multiple domains, including psychological health, sexual functioning, body image, and interpersonal relationships [85]. Women diagnosed with vaginal cancer often experience elevated levels of anxiety, depression, and psychological distress—frequently surpassing those observed in the general population. This distress tends to peak during periods of diagnostic uncertainty and at the initiation of treatment [86].

Treatment-related changes contribute to sexual dysfunction, reduced sexual desire, and dissatisfaction within intimate relationships [87]. These issues are commonly linked to physical symptoms such as pain and anatomical changes, as well as psychological factors, including shame, diminished self-confidence, and fear of intimacy [88]. Alterations in body image and a sense of “not being the same woman” are frequently reported, leading to decreased self-esteem and increased social withdrawal [89]. QoL is further diminished by the cumulative burden of physical symptoms such as pain, fatigue, and insomnia, alongside emotional and social challenges [90]. Additional stressors, such as financial hardship and disrupted social or familial roles, compound the difficulties faced by patients [91]. Importantly, psychological distress can adversely affect treatment adherence and recovery, highlighting the need for integrated mental health support within oncologic care to improve both emotional well-being and clinical outcomes [92].

### 4.8. Research Gaps and Future Directions

#### 4.8.1. Need for Prospective Trials and Molecular Studies

Vaginal adenocarcinoma is a rare and heterogeneous malignancy, comprising less than 10% of primary vaginal cancers. Due to its low incidence and histologic diversity—such as clear cell, endometrioid, and mucinous subtypes—clinical evidence remains limited. Current treatment strategies are largely extrapolated from cervical or endometrial cancer protocols or based on small retrospective series, leading to inconsistent management approaches and uncertain outcomes.

Prospective clinical trials are urgently needed to establish standardized treatment protocols and improve prognostication. Most existing data are derived from retrospective case series with variable staging methods, treatment regimens, and follow-up durations. Multicenter or international collaboration is crucial to conduct well-powered trials that clarify the roles of surgery, radiotherapy, chemotherapy, and targeted therapies, particularly in early-stage and recurrent disease. In addition, molecular and genomic studies are essential to deepen our understanding of vaginal adenocarcinoma pathogenesis and identify actionable targets. While clear cell adenocarcinoma has been associated with in utero DES exposure, the molecular drivers of non-DES-associated subtypes are poorly defined. Emerging data suggest overlaps with other Müllerian-derived tumors, but comprehensive profiling remains scarce. Studies focusing on gene mutations, pathway alterations, mismatch repair status, and immunologic markers could inform biomarker-driven clinical trials and personalized therapy. Furthermore, functional validation using organoids or xenografts and the integration of transcriptomic and epigenetic data may also enhance drug discovery and treatment optimization.

#### 4.8.2. Potential Biomarkers for Early Diagnosis

Alterations in the PI3K/AKT/mTOR pathway: The PI3K/AKT/mTOR pathway plays a crucial role in cancer development and progression, including gynecological malignancies [93,94]. This pathway regulates cell survival, growth, and proliferation, and its aberrant activation is common in various cancer types [93,95]. In ovarian cancer, the PI3K/AKT/mTOR pathway is significant for tumorigenesis and progression [95]. Inhibitors targeting this pathway show promise in preclinical and clinical studies for gynecological cancers, although none have yet received US Food and Drug Administration (FDA) approval for ovarian cancer treatment [94,95]. Some cancers exhibit high mTOR pathway activity without canonical genetic alterations, suggesting multiple mechanisms of activation [93]. Given the pathway’s importance in cancer biology and potential as a therapeutic target, further research into PI3K/AKT/mTOR inhibitors is warranted to improve the outcomes of gynecological cancers [94,96].

Mismatch repair deficiency: In the KEYNOTE-158 study, patients with non-colorectal, mismatch repair (MMR)-deficient cancers that had progressed despite conventional treatment were enrolled to receive anti-PD-1 immunotherapy [97]. The cohort encompassed twenty-seven different tumor types and included forty-nine cases of endometrial cancer, fifteen cases of ovarian cancer, six cases of cervical cancer, one case of vaginal cancer, and one case of vulvar cancer. Across the entire cohort, an objective response rate of 36.3% was observed, with a median overall survival of 23.5 months [97]. However, the response and progression-free survival rates of vaginal cancer were not reported. These findings suggest that immune checkpoint inhibitors may serve as an effective adjunct therapy for patients with mismatch repair-deficient gynecologic malignancies.

TP53 mutations: TP53 mutations and protein accumulation have been observed in vaginal adenocarcinomas, with varying frequencies. Skomedal et al. found TP53 alterations in 50% of primary vaginal carcinomas [98], whereas Lee et al. noted that TP53 mutations are rare in mesonephric adenocarcinomas [4]. Talia et al. reported intense p53 positivity in two cases of primary vaginal mucinous adenocarcinoma, suggesting TP53 mutation involvement [99]. However, Waggoner et al. found no p53 mutations in clear cell adenocarcinomas of the vagina and cervix, despite observing p53 protein overexpression in 67% of cases [100]. This overexpression was hypothesized to be a response to DNA damage rather than mutational inactivation. These studies highlight the complex role of TP53 in vaginal adenocarcinomas, with its involvement varying across different subtypes and potentially influencing tumor behavior and prognosis.

#### 4.8.3. Personalized Therapeutic Approaches

PIK3CA: PIK3CA has not been greatly studied within vaginal cancer. We, therefore, searched for the PIK3CA mutation in relation to nearby cervical and vulva cancers. PIK3CA mutations are prevalent in various gynecological cancers, including cervical adenocarcinoma and squamous cell carcinoma [101,102]. These mutations, particularly in hotspot locations, lead to hyperactivation of the PI3K/AKT/mTOR pathway, promoting oncogenesis [102]. PIK3CA mutations are associated with decreased progression-free and overall survival of cervical adenocarcinoma patients [101]. In squamous cervical carcinomas, PIK3CA mutations correlate with a higher tumor mutation burden and increased mutations in other cancer-associated pathways [102]. Targeting PIK3CA mutations with specific inhibitors, such as alpelisib, has shown promising clinical activity in advanced gynecological cancers, particularly in endometrial cancer patients [103]. These findings highlight the potential of PIK3CA mutations as both prognostic biomarkers and therapeutic targets in gynecological cancers, warranting further investigation in biomarker-driven clinical trials [101,103].

PTEN: PTEN has not been extensively studied in the field of vaginal cancer, and we searched for the PTEN mutations related to nearby cervical and vulva cancers. In cervical cancer, abnormal activation of the PI3K/AKT/mTOR pathway plays a key role in carcinogenesis, particularly in HPV-positive lesions [104]. Elevated expression levels of phosphorylated PI3K, AKT, and mTOR proteins have been observed in cervical cancer tissues compared to adjacent or preinvasive lesions. Under hypoxic conditions, mTORC1 signaling allows HPV-positive cells to evade senescence, further promoting tumor survival [105]. This suggests that the overactivation of the AKT/mTOR axis contributes to cervical cancer progression and resistance to cellular stress. Moreover, reduced PTEN expression correlates with an advanced stage, larger tumor size, and lymph node metastasis, while increased AKT/mTOR expression is associated with a worse prognosis [106]. In vulvar cancer, mTOR is frequently expressed and identified as a downstream effector of the AKT pathway [107]. In vitro studies demonstrate that mTOR inhibitors such as rapamycin, everolimus, and AZD2014 effectively suppress the proliferation of vulvar cancer cell lines [108].

Recent research has explored targeting PTEN loss in cancer therapy. PTEN deficiency enhances the sensitivity of cholangiocarcinoma to proteasome inhibitors, offering a potential treatment strategy [109]. In triple-negative breast cancer, a novel peptide–drug conjugate exploits enhanced albumin metabolism in PTEN-deficient cells to deliver chemotherapeutic payloads, showing promise against metastatic disease [110]. PI3Kβ inhibition with AZD8186 demonstrates efficacy in PTEN-deficient tumors, particularly when combined with chemotherapy or immunotherapy [111]. Post-translational modifications of PTEN, including acetylation, oxidation, phosphorylation, sumoylation, and ubiquitination, affect its stability, localization, and activity, representing potential therapeutic targets for tumors with at least one wild-type PTEN allele [112]. These studies highlight diverse approaches to targeting PTEN-deficient cancers, moving beyond conventional PI3K/AKT pathway modulation and offering new perspectives for treating aggressive and heterogeneous tumors

HER2: HER2 mutations occur in a small percentage of gynecologic malignancies, particularly in endometrial, ovarian, and cervical cancers [113,114]. HER2 mutations often involve the tyrosine kinase domain and are mutually exclusive with epidermal growth factor receptor (EGFR) and KRAS mutations [113,114]. While HER2 protein overexpression is not always present in HER2-mutated tumors, gene amplification is common [113]. Targeted anti-HER2 therapies have shown promise in clinical trials, with improved outcomes observed in patients receiving HER2-directed treatments [114]. These findings highlight the importance of testing for HER2 alterations in gynecologic cancers and suggest potential benefits of HER2-targeted therapies in this patient population.

Immunotherapy: A case of a young patient with recurrent vaginal clear cell carcinoma who achieved a complete and sustained response following treatment with pembrolizumab was reported [11]. Recurrent gynecological clear cell carcinoma (rGCCC, including vaginal cancer) responds poorly to chemotherapy, and emerging evidence suggests a potential synergistic effect of immune checkpoint inhibitors and anti-angiogenic therapy. The Determining Effects of Platelet Inhibition on Vaso-Occlusive Events (DOVE) phase 2 trial will evaluate the efficacy of dostarlimab with or without bevacizumab versus standard chemotherapy in 198 patients with rGCCC, with progression-free survival as the primary endpoint [115]. These studies highlight the potential roles of immune checkpoint inhibitors, alone or in combination with anti-angiogenic therapy, as promising therapeutic strategies for recurrent gynecological clear cell carcinoma, including rare vaginal cases.

## 5. Conclusions and Future Directions

### 5.1. Summary of Key Points

Due to its rarity, most of the evidence regarding vaginal cancer comes from case reports and small studies, emphasizing the need for continued reporting to expand knowledge about this uncommon malignancy [12]. Primary vaginal adenocarcinoma and rare vaginal malignancies encompass a wide spectrum of histological subtypes, including clear cell, mesonephric, endometrioid, intestinal-type, adenoid cystic, and enteric-type adenocarcinomas, as well as rare tumors like neuroendocrine carcinoma and cystadenocarcinoma. Patients range from young adults to elderly women, with common symptoms such as vaginal bleeding, pelvic pain, and mass formation. The diagnosis is frequently confirmed by imaging and immunohistochemistry, and the treatment strategies vary depending on the tumor type and stage, encompassing surgery, chemotherapy, radiotherapy, immunotherapy (e.g., pembrolizumab), and advanced techniques like fluorescence-guided surgery and proton beam therapy. Outcomes range from long-term remission to a poor prognosis, especially for advanced or aggressive cases. These cases underscore the diagnostic complexity and therapeutic challenges of vaginal malignancies, and highlight the importance of individualized, multidisciplinary management and ongoing reporting to improve evidence-based care.

### 5.2. Clinical Implications

The clinical implications of these cases highlight the need for heightened awareness and early recognition of rare vaginal malignancies, which often present with nonspecific symptoms such as bleeding or pelvic pain. An accurate histopathological diagnosis, supported by immunohistochemistry, is essential to differentiate primary vaginal adenocarcinomas from metastatic or secondary tumors. Given the diversity in histologic subtypes and variable treatment responses, a multidisciplinary approach is critical to optimize patient outcomes. Emerging therapies, including immune checkpoint inhibitors and precision radiotherapy, show promise, particularly for recurrent or treatment-resistant cases. These findings emphasize the importance of individualized treatment planning, a consideration of fertility and QoL in younger patients, and the inclusion of patients with rare tumor types in clinical trials to establish evidence-based management guidelines.

## Figures and Tables

**Table 1:** Search strategy outline.

Item	Specifications
Period	From January 2016 to 28 April 2025
Database	PubMed, Scopus, Web of Science
Search term used	Vaginal adenocarcinoma
Inclusion and exclusion criteria	All references were SCI-indexed articles written in English
Selection process	Two independent reviewers evaluated the titles and abstracts to determine eligibility

**Table 2:** Clinical and pathologic summary of reported cases of primary vaginal adenocarcinoma and rare vaginal malignancies (2016–2025).

Author, Year	Age	Clinical Presentation	Diagnosis	Management	Outcome
Zhang et al., 2019 [9]	45	Vaginal swelling, dyspnea, itching	Adenoid cystic carcinoma	Chemoradiotherapy	Alive at 13 months
Shen et al., 2022 [10]	64	Vaginal bleeding/discharge	Stage IVA squamous cell carcinoma	Chemo, radiotherapy, immunotherapy, TKIs	Complete remission
Porragas-Paseiro et al., 2023 [11]	38	Recurrent disease	Clear cell carcinoma	Pembrolizumab	Complete, durable response
Ferrari et al., 2023 [12]	52	Vaginal bleeding	Mesonephric adenocarcinoma	Surgery, adjuvant therapy	Mean follow-up 6 years, mostly favorable
Yang et al., 2019 [13]	57	Vaginal bleeding, pelvic pain	Endometrioid adenocarcinoma	Surgery, chemotherapy	No recurrence at 12 months
Mu et al., 2023 [14]	40	Incessant menstruation, distension	Endometrioid adenocarcinoma	Staged surgery, chemo (6 cycles)	No recurrence at 2 years
Saijilafu et al., 2024 [15]	62	Postmenopausal bleeding	Stage IIB adenocarcinoma	Chemo + external + intracavitary radiotherapy	Tumor control at 3 months
Barcellini et al., 2021 [16]	80	Recurrent vaginal tumor	Squamous cell carcinoma	Proton beam therapy	Complete response at 12 months
Pang et al., 2019 [5]	39	Chronic vaginal pain, bleeding	Clear cell carcinoma (adenosis origin)	Wide excision + chemoradiotherapy	No recurrence at 16 months
Plesinac-Karapandzic et al., 2017 [17]	22	Vaginal mass	Clear cell/mesonephric carcinoma	Radiotherapy chemotherapy	Disease-free at 11 years, morbidity noted
Kumar et al., 2022 [18]	40	Post-hysterectomy bleeding	Mesonephric carcinoma	IHC-based diagnosis	Not stated
Li et al., 2023 [19]	55	Vaginal bleeding	Clear cell carcinoma (rectovaginal septum)	Surgery + chemotherapy	Complete response
Haddout et al., 2022 [20]	60	Inguinal mass, vaginal lesion	Clear cell carcinoma	Surgery + chemoradiotherapy	Poor prognosis
Felicelli et al., 2023 [21]	63	Vaginal polypoid mass	HPV-associated enteric-type adenocarcinoma	Surgery	First reported case, outcome not detailed
Sabri et al., 2022 [7]	62	Vaginal tumor	Intestinal-type adenocarcinoma	Varied treatments	Prognosis depends on multiple factors
Ugwu et al., 2019 [22]	40	Vaginal mass, bleeding	Intestinal-type adenocarcinoma	Surgery	Recognition key for diagnosis
Mei et al., 2020 [23]	40	Irregular bleeding	Clear cell carcinoma + HWW syndrome	Surgery	Not stated
Warembourg et al., 2016 [24]	63	Pelvic pain	Cystadenocarcinoma (rectovaginal)	Surgery + chemotherapy	No recurrence at 36 months
Kalampokas et al., 2023 [25]	79	Vaginal pressure feeling and bleeding	Small-cell neuroendocrine carcinoma	Surgery	-
Nguyen-Xuan et al., 2021 [26]	44	Metrorrhagia	Clear cell carcinoma	Fluorescence-guided surgery	Negative margins
Lei & Zhang, 2024 [27]	40	Irregular bleeding	Clear cell carcinoma + HWW syndrome	Radical surgery, chemotherapy	Lung metastasis at 4 years

TKIs, tyrosine kinase inhibitors; HWW, Herlyn–Werner–Wunderlich syndrome.

**Table 3:** Key signs and symptoms.

Symptom	Description	Frequency/Notes	References
Abnormal Vaginal Bleeding	Includes postmenopausal, postcoital, or intermenstrual bleeding	Most common symptom (50–75% of cases)	[10,45]
Vaginal Discharge	Persistent or foul-smelling discharge; may suggest tumor necrosis or infection	Common	[10,46]
Palpable Mass or Lump	Nodule or thickening, especially in upper third of the vaginal wall	Frequently observed on a physical exam	[17]
Pain or Discomfort	Pelvic pain, dyspareunia, rectal pain; more common in advanced stages	Variable	[24,46]
Other Symptoms	Itching, burning, urinary symptoms (dysuria, hematuria), constipation, hematuria (if local invasion occurs)	Less common, dependent on tumor spread	[26]

**Table 4:** IHC profiles and differential diagnosis of vaginal adenocarcinoma.

Tumor Type	Positive Markers (Fold Change vs. Non-Relevant Tumors)	Negative Markers (Reduction vs. Relevant Tumors)	Diagnostic Utility
Intestinal-Type Vaginal ADC	SATB2 (2–5×), CDX2 (3–10×), CK20 (5–20×), CEA (2–4×)	CK7 (>90% ↓), PAX8 (near-absent), p16 (50–80% ↓)	SATB2/CDX2 confirm an intestinal origin; CK7/PAX8 exclude gynecologic primary tumors.
Endometrial/Ovarian ADC	PAX8 (5–50×), CK7 (10–30×), ER/PR (2–10×)	SATB2 (>95% ↓), CK20 (>80% ↓)	PAX8/CK7 + ER/PR confirm a Müllerian origin; SATB2 excludes CRC.
Cervical ADC	p16 (10–100×), CEA (2–5×)	ER/PR (>90% ↓), Vimentin (>70% ↓)	p16’s extreme overexpression indicates an HPV-driven etiology.
Serous Papillary ADC	PAX8/WT1 (5–20×), p53 (10–50×), CA125 (↑ ↑ serum)	SATB2/CK20 (>95% ↓)	An aberrant WT1/p53 pattern confirms a serous subtype.

↓ reduction, ↑ increase.

**Table 5:** Comparison of staging systems for vaginal cancer.

AJCC Stage	Stage Grouping (TNM)	FIGO Stage	Stage Description
IA	T1a	I	Present only in the vagina and is no larger than 2.0 cm (4/5 inch) (T1a)
	N0		No spread to nearby lymph nodes (N0) or to distant sites (M0)
IB	T1b	I	Present only in the vagina and is larger than 2.0 cm (4/5 inch) (T1b)
	N0		No spread to nearby lymph nodes (N0) or to distant sites (M0)
IIA	T2a	II	Cancer has grown through the vaginal wall, but not as far as the pelvic wall, and is no larger than 2.0 cm (4/5 inch) (T2a)
	N0		No spread to nearby lymph nodes (N0) or to distant sites (M0)
IIB	T2b	II	Cancer has grown through the vaginal wall, but not as far as the pelvic wall, and is larger than 2.0 cm (4/5 inch) (T2b)
	N0		No spread to nearby lymph nodes (N0) or to distant sites (M0)
III	T1 to T3	III	Cancer can be any size and might be growing into the pelvic wall, and/or growing into the lower one-third of the vagina, and/or has blocked the flow of urine (hydronephrosis), which is causing kidney problems (T1 to T3); has spread to nearby lymph nodes in the pelvis or groin (inguinal) area (N1) but not distant sites (M0)
	N1		
	OR		
	T3	III	Cancer is growing into the pelvic wall, and/or growing into the lower one-third of the vagina, and/or has blocked the flow of urine (hydronephrosis), which is causing kidney problems (T3)
	N0		No spread to nearby lymph nodes (N0) or to distant sites (M0)
IVA	T4	IVA	Cancer is growing into the bladder or rectum or is growing out of the pelvis (T4)
	Any N		May/may not have spread to lymph nodes in the pelvis or groin (inguinal area) (Any N); has not spread to distant sites (M0)
IVB	Any T	IVB	Cancer has spread to distant organs such as the lungs or bones (M1). It can be any size and might or might not have grown into nearby structures or organs (Any T)
	Any N		May/may not have spread to nearby lymph nodes (Any N)
	M1		May/may not have spread to nearby lymph nodes (Any N)

## Data Availability

All data are included in the manuscript.

## Abbreviations

The following abbreviations are used in this manuscript:

CT	Computed tomography
DFS	Disease-free survival
EBRT	External beam radiation therapy
EGFR	Epidermal growth factor receptor
ER	Estrogen receptor
FDA	Food and Drug Administration
FIGO	Federation Internationale de Gynecologie et d’Obstetrique
HDR	High dose rate
MRI	Magnetic resonance imaging
NCDB	National Cancer Data Base
PET	Positron emission tomography
PR	Progesterone receptor
QoL	Quality of life
